# Optical Characterization of ALD-Coated Nanoporous Alumina Structures: Effect of Sample Geometry or Coated Layer Material

**DOI:** 10.3390/mi14040839

**Published:** 2023-04-12

**Authors:** Ana Laura Cuevas, Víctor Vega, Antonia Domínguez, Ana Silvia González, Víctor M. Prida, Juana Benavente

**Affiliations:** 1 Unidad de Nanotecnología, SCBI Centro, Universidad de Málaga, E-29071 Málaga, Spain; 2 Laboratorio de Membranas Nanoporosas, Servicicios Científico-Técnicos, Universidad de Oviedo, E-33006 Oviedo, Spain; 3 Departmento de Física, Facultad de Ciencias, Universidad de Oviedo, E-33007 Oviedo, Spain; 4 Departmento de Física Aplicada I, Facultad de Ciencias, Universidad de Málaga, E-29071 Málaga, Spain

**Keywords:** nanoporous alumina structures, ellipsometric spectroscopy, atomic layer deposition, photoluminescence, optical properties

## Abstract

Optical characterization of nanoporous alumina-based structures (NPA-bSs), obtained by ALD deposition of a thin conformal SiO_2_ layer on two alumina nanosupports with different geometrical parameters (pore size and interpore distance), was performed by two noninvasive and nondestructive techniques such as spectroscopic ellipsometry (SE) and photoluminescence (Ph) spectra. SE measurements allow us to estimate the refraction index and extinction coefficient for the studied samples and their dependence with wavelength for the 250–1700 nm interval, showing the effect of sample geometry and cover-layer material (SiO_2_, TiO_2_, or Fe_2_O_3_), which significantly affect the oscillatory character of both parameters, as well as changes associated with the light incidence angle, which are attributed to surface impurities and inhomogeneity. Photoluminescence curves exhibit a similar shape independently of sample pore-size/porosity, but they seem to affect intensity values. This analysis shows the potential application of these NPA-bSs platforms to nanophotonics, optical sensing, or biosensing.

## 1. Introduction

Atomic layer deposition (ALD), also known as atomic layer epitaxy, is a nanofabrication technique especially suited to grow thin film coatings onto complex substrates. Furthermore, as a consequence of its self-limiting deposition nature, ALD displays other remarkable features that are unique to this technique, such as atomic control on the thickness of the deposited layers and the possibility of growing nanolaminates or stacks of different material layers [1,2,3]. Therefore, ALD is usually considered a promising technique with huge prospects in the fields of micro- and nanofabrication technologies [3,4,5]. In addition to that, the combined use of ALD with either soft or hard templates opens up a new route for the development of novel nanomaterials and nanodevices through the so-called atomic layer assembly fabrication method [6,7,8]. These advanced nanomaterials have found promising applications in fields such as catalysis [9,10,11], energy conversion and storage [12,13,14,15,16,17,18,19], or photonics [7,8,20]. For these applications, the precise control of the deposited materials thickness and composition, which can be achieved by atomic layer deposition techniques, becomes a key enabling factor.

On the other hand, nanoporous alumina structures (NPASs) obtained by electrochemical anodization of aluminum foils using the two-step anodization method [21,22] are nowadays systems of great interest due to their structural regularity (cylindrical nanopores without tortuosity and very narrow pore size distribution) and large specific surface area [23,24]. A significant characteristic of this fabrication process is the easy modification of NPAS geometrical parameters by simple variation of the anodization conditions used during the first step (electrolyte solution, applied voltage, and/or temperature), which allows samples with average pore sizes ranging between 20 and 400 nm and an interpore distance between 40 and 450 to be obtained [25,26]. NPASs were initially used in the fabrication of nanorows, nanowires, and membranes for separation of heavy metal cations and drug delivery due also to their good chemical/thermal stability and hardness, although lately they have also been considered for catalysis, optoelectronics, and sensing applications [26,27,28,29,30], but the photonic crystal character associated with their regular nanostructure was already reported [31,32]. In this context, the effect of sample porosity and pore size on optical characteristic parameters such as refraction index, extinction coefficient, and/or dielectric constant has also been considered lately [33,34]. Other characteristics such as photoluminescence or solar absorptivity have also been reported, opening the use of NPASs as sensors, light-emitting diodes, and solar cells [35,36,37,38]. In this context, as it was already reported [39,40,41], surface coating of the NPASs by one or two layers of different ceramic oxides (SiO_2_, Fe_2_O_3_, TiO_2_, ZnO, …) by the ALD technique has permitted the modification of the geometrical, ionic transport, and optical parameters of these nanoporous alumina-based structures (NPA-bSs). On the other hand, both electrochemical anodization and ALD coating processes might provoke the presence of impurities on sample surfaces as well as the increase of its roughness (coalescent effect associated with cover layer formation [42]), which can have an influence on the values of different optical parameters of these NPA-bSs, and consequently, information related to such effects is necessary. Another point of interest is the influence of the ALD coating layer on the photoluminescence character of the NPASs, which seems to depend on several factors that are related to sample fabrication and affect the pore size/porosity of the samples [43], since it could be useful in some applications of NPA-bSs (sensing and biosensing systems [44]).

Different techniques can be used for the optical characterization of thin films [45,46,47], with spectroscopy ellipsometry (SE) measurements being one of the most surface-sensitive optical tools for the characterization of substrates and overlayers used for different sensor applications [48,49,50]. SE is a precise and accurate surface technique for characterizing substrates and overlayers, commonly used for the determination of basic optical parameters of thin films such as the refractive index (n) and the extinction coefficient (k), two parameters related to the interaction between materials and incident light and associated with refraction or absorption, respectively, as well as the dielectric constant (real (ε_r_) and imaginary (ε_i_) parts). The knowledge of optical constants provides the fundamental basis for designing and manufacturing optical devices, but morphological/structural (layer thickness or roughness) information can also be obtained by modeled analysis of the SE data [51,52]. In this context, SE is nowadays commonly used for the characterization of coatings in different fields (materials protection, photovoltaic materials, flat panel display devices, biological layers, biofilms, etc.) [53,54].

In this work, the effect of geometrical characteristics (pore size and porosity) on the optical behavior of two NPA-bSs samples, obtained by coating them with a SiO_2_ layer (by the ALD process), two NPASs with different geometrical parameters, are analyzed. As it is well known, silicon dioxide is a remarkably interesting material for surface modification due to its high chemical and thermal stability as well as its dielectric nature, and ALD of SiO_2_ layers offers exceptional deposition conformality for nanoporous alumina structures with a high aspect ratio [55]. Optical characterization was performed by: (i) spectroscopic ellipsometry (SE) measurements at three different light incident angles for wavelengths covering the visible and near infrared (NIF) optical regions; and (ii) photoluminescence spectra with a 325 nm excitation laser. Moreover, the effect of surface material on refractive index and extinction coefficient values as well as on the photoluminescence character of the samples was also considered by comparing the results obtained for the SiO_2_-covered sample with those for other NPA-bSs with similar pore size and porosity but different ALD coating layers (TiO_2_ or Fe_2_O_3_). The results obtained can be of interest for optical applications of nanoporous ceramic samples.

## 2. Materials and Methods

### 2.1. Materials

Aluminum discs (0.5 mm in thickness and 25 mm in diameter) of high purity (Al 99.999%, Goodfellow, UK), previously cleaned with isopropanol and ethanol and electropolished in a 1:3 vol. solution of perchloric acid in ethanol, were employed as substrates for the fabrication of NPA-bSs samples by the two-step anodization process. The anodization process employed a solution of oxalic acid (0.3 M) as the electrolyte and 40 V for the anodization voltage [21,22,56], and the first anodization step was performed during 24 h at 0–1 °C; after this process, an acidic solution of CrO_3_ and H_3_PO_4_ at 35 °C for 24 h was used for removing the aluminum oxide layer. Then, the second anodization step, under the same anodization conditions, was carried out by adjusting the time until the NPAS achieved a thickness of ~60 µm; an aqueous mixture of HCl and CuCl_2_ was employed in order to remove an area of around 1 cm^2^ of the remaining Al at the NPAS’s underside. Furthermore, we employed H_3_PO_4_ 5% aqueous solution at room temperature to remove the alumina barrier layer that blocks the pores bottom. The time for this wet chemical etching was 90 min. Finally, we carried out a widening of the pores through chemical etching in an H_3_PO_4_ 5% aqueous solution at 30 °C in order to adjust the initial pore diameter of 35 nm to the desired value for the specific samples.

Once we have synthesized the NPASs, ALD coating of the samples was performed in a Savannah 100 thermal ALD reactor (Cambridge Nanotech, Waltham, MA, USA), using exposure mode, in which the gaseous precursors are let stay in the reaction chamber for prolonged times, thus allowing them to diffuse along the high aspect ratio pore channels of NPAS. Different precursors (3-Aminopropyl)triethoxysilane, titanium tetraisopropoxide, and ferrocene), were used for the deposition of the different conformal coating layers (SiO_2_, TiO_2_, and Fe_2_O_3_, respectively) [40,41], and Ar was also used as a purge and carrier gas. Furthermore, we employed different oxidant agents (O_3_ or H_2_O), depending on the specific chemistry of the ALD precursor used for the functionalization of the NPA-bSs. The time and temperature were different depending on the precursor employed in the ALD processes for the different coating materials. The selected precursor temperature, pulse time durations, and experimental conditions are indicated in Table 1. For all processes, an Ar flow of 50 sccm was selected between two subsequent precursor pulses during 90 s in order to remove the excess reactants and resulting by-products of the ALD process. Finally, we had to take into account the different growth rates of each metal oxide, and we accordingly selected the number of ALD cycles for the different processes to adjust the thickness of the deposited layer to around 4–5 nm [57].

### 2.2. Surface Analysis

Scanning electron microscopy (SEM) images of the top view of the different NPA-bSs studied in this work were obtained in a tungsten-filament SEM (JEOL-5600 Akishima, Tokyo, Japan) operated at 20 kV. Due to the electrically isolating character of the NPA-bSs, the samples were sputtered with a thin conductive gold layer. Image analysis was employed to extract the average values of pore diameter, interpore distance, and porosity from the SEM images, using for that purpose ImageJ (v 1.53t) software [58].

### 2.3. Optical Characterizations

Spectroscopic Ellipsometry (SE) measurements were carried out for wavelengths ranging between 250 nm and 1700 nm with unsupported samples using a goniometer spectrometer (Sopra-Semilab GES-5E, Paris, France), which allows the automatic selection of measurements angles; three different angles (65°, 70°, and 75°) were selected. WinElli software from Sopra-Semilab (Paris, France) was used for data analysis and fittings.

**Figure 1:**
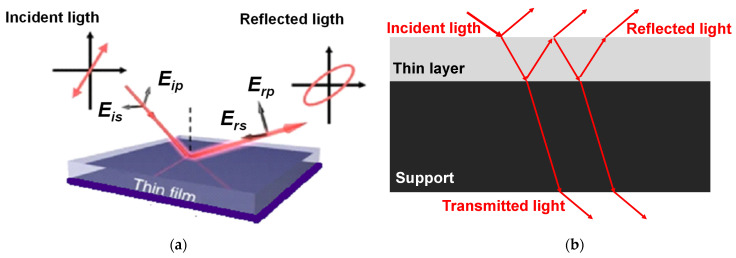
Scheme of: (**a**) polarization states of incident light and after reflection from a surface; (**b**) incident, reflected, and transmitted light for a supported thin layer.

Spectroscopic ellipsometry is a contactless technique used to characterize single layers, or a small number of well-defined layers, as optically homogeneous and isotropic. SE measures the change in the polarization state of light after reflection from a sample surface through the so-called ellipsometric angles, ψ and Δ, related to the amount of reflected light polarized in the perpendicular plane with respect to the incidence light plane (rp) and the amount of reflected light polarized in a plane parallel to the incidence light plane (rs), as it is schematically indicated in Figure 1a. These parameters are related by the following expression [51,59,60,61]:

tan(Ψ)·e^iΔ^ = r_p_/r_s_ (1)

where tan(Ψ) is the amplitude ratio upon reflection and Δ is the phase shift difference [61]. Figure 1b shows the scheme of light reflection for a two-layer sample.

The Photoluminescence (PL) character of the studied samples was measured with a photoluminescence microscope from HORIBA Scientific (LabRam PL Microscope, Kyoto, Japan), at room temperature, using a 325 nm laser as an excitation light source with a beam power of 0.2 mW in the range from 325 to 700 nm.

## 3. Results and Discussion

### 3.1. SEM Characterization

**Figure 2:**
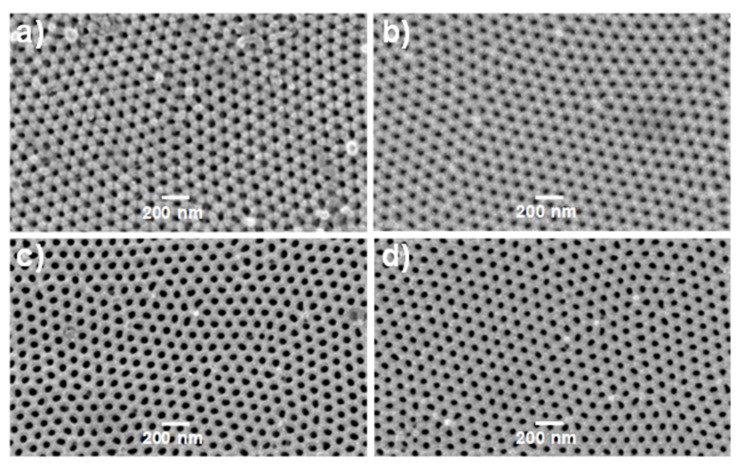
SEM top surface images of samples (**a**) Ox(A), (**b**) Ox(A)/SiO_2_, (**c**) Ox(B), and (**d**) Ox(B)/SiO_2_.

Figure 2 displays SEM micrographs of the top surfaces of both NPASs and NPAS/SiO_2_ samples, showing a high ordering degree of the nanopore arrangement and their nearly non-disperse pore size distribution. From these images, pore diameter values were obtained through computer-assisted image analysis [58]. The images in Figure 2a,b correspond to samples of Ox(A) before and after applying a SiO_2_ coating by ALD (Ox(A)/SiO_2_ sample), evidencing a reduction in the average value of pore diameter from 34 to 22 nm, which corresponds to the thickness of the ALD SiO_2_ layer deposited into the pores of the nanoporous alumina membrane. A similar percentage reduction in pore diameter after pores were covered with a SiO_2_ layer can be observed for samples Ox(B) (53 nm diameter) and Ox(B)/SiO_2_ (42 nm pore diameter) in Figure 2c and d, respectively, thus evidencing the reliability and conformality of SiO_2_ layers deposited by ALD.

### 3.2. Optical Characterization

Optical techniques for the characterization of thin films, such as spectroscopic ellipsometry (SE) or photoluminescence (Ph), are of great importance since they are non-destructive measurements and do not need vacuum environments, and they provide significant information on important parameters or characteristics of the analyzed samples. In addition to the use of SE for the estimation of thin samples thickness, information on superficial modifications (impurities and/or roughness [62,63]) is also of interest since it could affect optical parameters.

**Figure 3:**
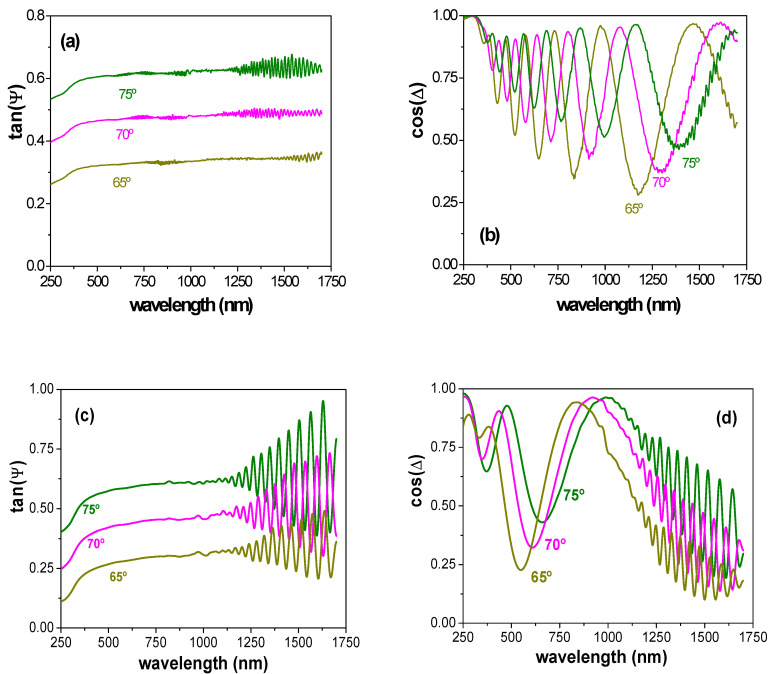
Wavelength dependence for tan(Ψ ) and cos(∆) at three light incident angles: 65° (dark yellow lines), 70° (magenta lines), and 75° (green lines). (**a**,**b**): Ox(A)/SiO_2_ sample; (**c**,**d**): Ox(B)/SiO_2_ sample.

Figure 3 shows a comparison of wavelength dependence for both SE experimental parameters, tan(Ψ ) and cos(∆), for the two studied samples (Ox(A)/SiO_2_ and Ox(B)/SiO_2_) at three light incident angles (ϕ_0_ = 65°, 70°, or 75°), where differences depending on both sample geometry (pore-size/porosity) and incident angle can be observed. The main differences between both samples in the results shown in Figure 3 are the high number of oscillations exhibited by cos(∆) for the Ox(A)/SiO_2_ sample in both visible and NIF regions, independently of incident angle, while only small differences between both samples were obtained for tan(Ψ) values. However, it should be indicated that recorded values for the Ox(B)/SiO_2_ sample present very high dispersion for wavelengths higher than 1000 nm, and smoothed values for the 1000–1700 nm interval are indicated in Figure 3c,d, which could be related to the high transmittance of the sample for such a wavelength interval, but recorded values (without any manipulation) are presented as Supplementary Information (Figure S1).

**Figure 4:**
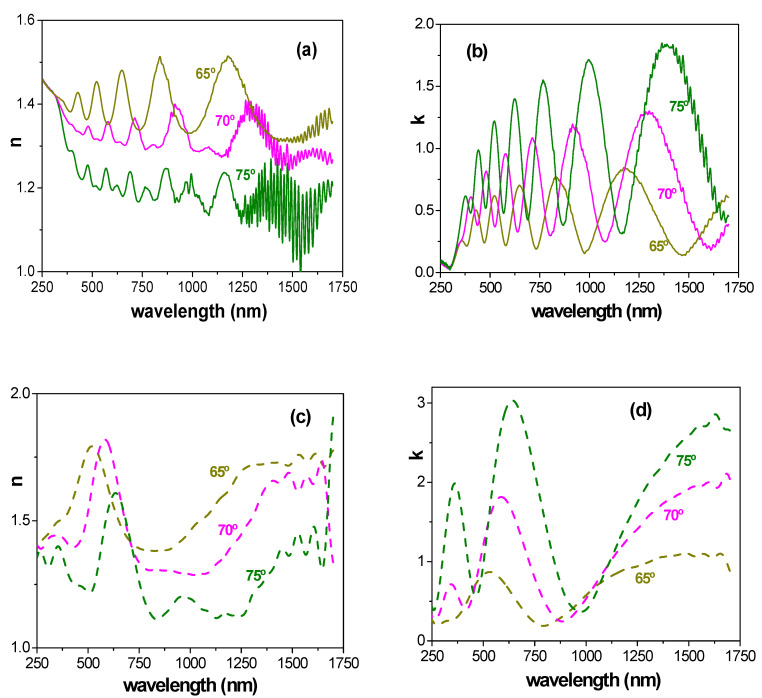
Wavelength dependence for the refraction index (n) and the extinction coefficient (k) at the three light incident angles: 65° (dark yellow lines), 70° (magenta lines), and 75° (green lines). (**a**,**c**): refraction index; (**b**,**d**): extinction coefficient. Solid lines: Ox(A)/SiO_2_ sample; dashed lines: Ox(B)/SiO_2_ sample.

Optical characteristic parameters such as the refractive index (n) and the extinction coefficient (k) can be determined from SE measurements using the ellipsometer software, and wavelength dependence for sample Ox(A)/SiO_2_ at the three light incident angles is shown in Figure 4a,b, respectively, while the variation of n and k with wavelength for the Ox(B)/SiO_2_ sample is shown in Figure 4c,d. As it can be observed, both samples exhibit wavelength oscillations, a characteristic of photonic crystals, with that effect being more significant for the Ox(A)/SiO_2_ sample, in agreement with its lower pore radii according to previous results [34]. This oscillatory behavior found in semiconducting and dielectric thin films has been theoretically modeled by Bao and Xu [64], although the complex morphology of NPA-bSs makes it difficult to apply to our system. On the other hand, a reduction in the values of the refraction index with the increase of the incident angle was obtained, which is attributed to the inhomogeneity and impurity of the top layer of the samples [41,65]. This latter point was already established by depth profile X-ray photoelectron spectroscopy (XPS) analysis (a destructive technique) [40,41], and it is mainly associated with the coating layer process (use of H_2_N(CH_2_)_3_Si(OC_2_H_5_)_3_ as a precursor for these samples), since those results indicate a high atomic concentration percentage of carbon on the sample surface and its reduction by around 50% after 0.6 sec of argon etching. In this context, the variation of the atomic concentration percentages of the different elements detected on the surface of an Ox/SiO_2_ sample with the XPS sputtering time is presented as Supplementary Information (Figure S2).

**Figure 5:**
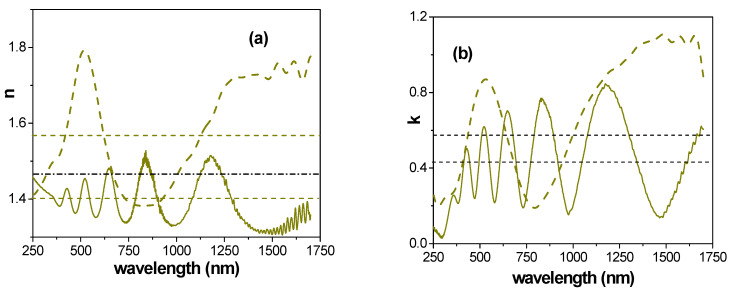
Comparison of wavelength dependence for the refraction index (**a**) and the extinction coefficient (**b**) at a light incident angle of 65°. Solid lines: Ox(A)/SiO_2_ sample; dashed lines: Ox(b)/SiO_2_ sample. Dark yellow horizontal lines indicate the average (<n> and <k>) values for each sample; a black horizontal dashed-dot line indicates the tabulated value of SiO_2_ refractive index (at λ = 632.8 nm).

The effect of sample geometry on n and k values can be more clearly observed in Figure 5, where a comparison of the wavelength dependence of both parameters for Ox(A)/SiO_2_ and Ox(b)/SiO_2_ samples at a light incident angle of 65° is presented and clear differences in both curve shape and values can be observed. Dark yellow horizontal dashed lines in Figure 5 indicate average values for each sample and parameter, with both the n and k values determined for the Ox(B)/SiO_2_ sample being higher than those obtained for the Ox(A)/SiO_2_, but the black horizontal dashed-dot line in Figure 5a corresponds to the tabulated value (at 632.8 nm) of SiO_2_ refractive index [66]. Moreover, since the dielectric constant is related to n and k: ε = (n + i·k)^2^, the wavelength dependence of ε_r_ and ε_i_ can also be determined, and they are shown as Supplementary Information (Figure S3).

**Figure 6:**
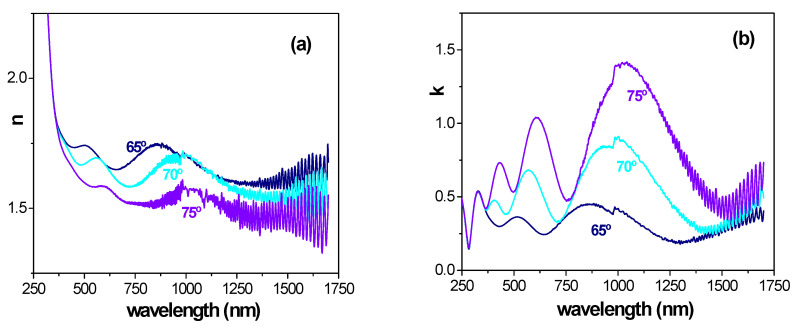

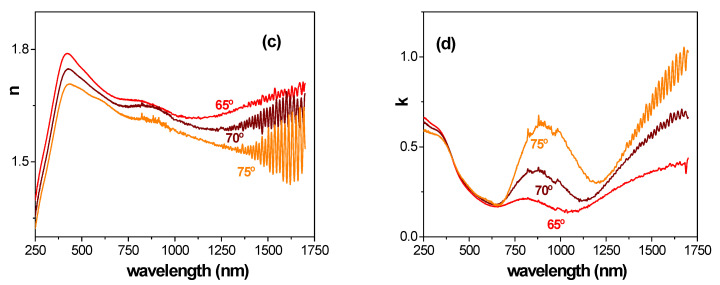
Effect of an Ox(A) support coated layer on refractive index (n) and extinction coefficient (k) for wavelengths ranging between 250 nm and 1700 nm determined at three different light incident angles. (**a**,**b**): Ox(A)/TiO_2_ sample; (**c**,**d**): Ox(A)/Fe_2_O_3_ sample.

The effect of cover-layer material for samples with similar geometrical parameters was also considered by comparing the wavelength dependence of n and k values determined for the Ox(A)/SiO_2_ sample with those obtained for the Ox(A) alumina support coated by ALD with a TiO_2_ or a Fe_2_O_3_ layer (Ox(A)/TiO_2_ or Ox(A)/Fe_2_O_3_ samples), and the obtained results are shown in Figure 6a,b for the Ox(A)/TiO_2_ sample and Figure 6c,d for the Ox(A)/Fe_2_O_3_. As expected, differences in the values of the refraction index and extinction coefficient depending on the ceramic oxide coating layer were obtained, but also in the shape of the wavelength dependence, reducing significantly for both materials the oscillatory character of the refraction index as well as the effect of the incident angle.

**Figure 7:**
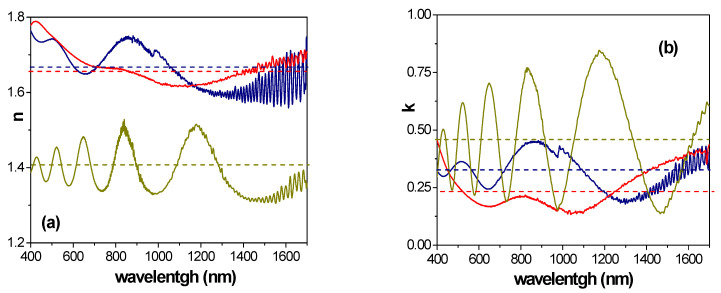
Comparison of wavelength dependence for the refraction index (**a**) and the extinction coefficient (**b**) at a light incident angle of 65° for the Ox(A)/SiO_2_ sample (dark yellow line), the Ox(A)/TiO_2_ sample (blue line), and the Ox(A)/Fe_2_O_3_ sample (red line). Horizontal dashed lines indicate average <n> or <k> values for each sample.

A comparison of wavelength dependence (400–1700 nm interval) for n and k values determined at 65° for Ox(A)/SiO_2_, Ox(A)/TiO_2_, and Ox(A)/Fe_2_O_3_ samples is presented in Figure 7, and from these results the following average values for both parameters (horizontal dashed lines in Figure 7) were obtained: <n(Ox(A)/SiO_2_)> = 1.40; <n(Ox(A)/TiO_2_)> = 1.67; <n(Ox(A)/Fe_2_O_3_)> = 1.66; <k(Ox(A)/SiO_2_)> = 0.47; <k(Ox(A)/TiO_2_)> = 0.33; and <k(Ox(A)/Fe_2_O_3_)> = 0.24. Taking into account the similarity in geometrical parameters of these samples, modifications for different optical parameters (n, k, as well as band gap, as determined by light transmission measurements [40]) are associated with the cover layer.

As it was already indicated, a particular property of nanoporous alumina structures (NPASs) obtained by the electrochemical anodization method is photoluminescence (PL), which was initially reported in the 1980s and is ascribed to structural defects, namely oxygen vacancies, present in NPASs as a consequence of the incorporation of impurities during the anodic oxidation process [35]. Since PL can be of interest for different applications, a number of papers correlating PL spectra with sample fabrication parameters (mainly anodization voltage but also electrolyte solutions and electrolyte mixtures or temperature, which affects sample nanometric geometry) or even aluminum purity have been reported lately [67,68,69,70,71]. These results show PL curves with similar shapes and maximum intensity wavelengths ranging between 420 nm and 460 nm, depending on anodization voltage values using oxalic acid as the electrolyte (for excitation wavelengths around 330 nm). However, in the case of malonic acid, different curve shapes and maximum intensity wavelength values (between 450 nm and 520 nm) were obtained, whereas aluminum impurity only seems to reduce the intensity of the curves. Consequently, the photoluminescence character of the NPA-bSs and the possible effect of sample geometry and even cover layer material on PL results have also been considered. In fact, photoluminescence for different kinds of SiO_2_-based films (nanotextured SiO_2_ and silicon oxynitride films, SiO_2_-coated ZnO nanoparticles, or SiO_2_ layers on Si support) as well as for a layered nanostructure (multi-walled carbon nanotube-TiO_2_ nanofiber composite), as a result of surface oxygen vacancies and other defects, have also been reported [71,72,73,74,75,76,77]. Therefore, an additional method to tune the intensity of PL is the use of appropriate coatings, which can have an active or passive role in PL effects.

**Figure 8:**
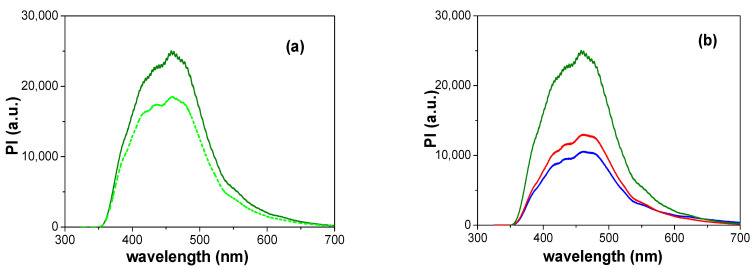
Photoluminescence spectra obtained for: (**a**) the Ox(A)/SiO_2_ sample (solid line) and the Ox(B)/SiO_2_ sample (dashed line); (**b**) the Ox(A)/SiO_2_ sample (green line), the Ox(A)/TiO_2_ sample (blue line), and the Ox(A)/Fe_2_O_3_ sample (red line).

Figure 8a shows a comparison of the photoluminescence spectra obtained for Ox(A)/SiO_2_ and Ox(B)/SiO_2_ samples using an excitation wavelength of 330 nm, and, as can be observed, the increase in sample pore-size/porosity does not modify the shape of the curve, with a maximum at a wavelength around 460 nm. In fact, the shape of the curves is similar to that obtained for SiO_2_-Si structures excited at 350 nm [76], as well as for high-purity alumina films (also obtained with 0.3 oxalic acid and deposited on soda-lime glass substrates [75]), when excited at 320 nm or 330 nm, but in that case the maximum intensity was at a wavelength of 400 nm or 410 nm. Moreover, the effect of coating-layer material on the photoluminescence spectrum for the NPA-bSs with similar geometry but different coating layers (SiO_2_, TiO_2_, or Fe_2_O_3_) is shown in Figure 8b, where the most significant difference is the intensity of the PL spectra depending on the cover layer material, with the following tendency: Ox/SiO_2_ >> Ox/Fe_2_O_3_ > Ox/TiO_2_, which indicates that both Fe_2_O_3_ and TiO_2_ have a passivating effect, thus substantially decreasing PL effects. These results support the possible use of the studied samples in bio-imaging, diagnostics, and sensing devices.

## 4. Conclusions

The optical characterization of nanoporous alumina structures coated with different ceramic oxides by ALD technique, performed in this study by spectroscopy, ellipsometry, and photoluminescence measurements, clearly shows the influence of sample geometry and coating material on the value of a characteristic optical parameter such as the refraction index of the samples as well as on its oscillatory behavior; both factors also seem to affect the photoluminescence behavior of the studied samples. Moreover, surface inhomogeneity and the presence of impurities associated with sample fabrication seem also to affect the values of optical parameters, according to the SE results obtained at different light incidence angles. On the other hand, this analysis indirectly supports the suitability of the ALD technique for functional modification of nanoporous structures, showing the potential application of these NPA-bSs platforms to nanophotonics, optical sensing, or biosensing.

## Figures and Tables

**Table 1:** Times and temperatures employed in the ALD processes for the different coating materials, where t_1_ is the precursor pulse time; t_2_ is the exposition time; and t_3_ is the purge lapse. For each material, the times used in the exposure to each precursor are shown by columns, whereas the precursor and substrate temperatures are indicated by lines.

Material (Substrate Temperature)	Precursors (Precursor Temperature)	t_1_ (s)	t_2_ (s)	t_3_ (s)
SiO_2_ (150 °C)	H_2_O (60 °C)	1	60	120
	O_3_ (25 °C)	0.1	60	120
	H_2_N(CH_2_)_3_Si(OC_2_H_5_)_3_ (100 °C)	2	60	120
TiO_2_ (200 °C)	H_2_O (60 °C)	1	60	120
	Ti[OCH(CH_3_)_2_]_4_ (75 °C)	1	60	60
Fe_2_O_3_ (230 °C)	O_3_ (25 °C)	0.1	60	120
	C_10_H_10_Fe (100 °C)	3	60	120

## Data Availability

Not applicable.

## Supplementary Materials

The following supporting information can be downloaded at: https://www.mdpi.com/article/10.3390/mi14040839/s1, Figure S1: Wavelength dependence for tan(Ψ) and cos(∆) at three light incident angles: 65° (dark yellow lines), 70° (magenta lines), and 75° (green lines) for sample Ox(B)/SiO_2_. Without applying data refinement (smoothing); Figure S2: Variation of atomic concentration percentages of the different elements detected on the Ox/SiO_2_ sample surface with the XPS sputtering time; Figure S3: Wavelength dependence of the dielectric constant (real part (a) and imaginary part (b)) for Ox(A)/SiO_2_ sample (solid line) and Ox(B)/SiO_2_ sample (dashed line) determined for a light incident angle of 65°.
