# Supplementary material for: Optical Characterization of ALD-Coated Nanoporous Alumina Structures: Effect of Sample Geometry or Coated Layer Material
Source: Micromachines (Basel). 2023 Apr 12;14(4):839. doi: 10.3390/mi14040839 (PMC10143564; doi:10.3390/mi14040839)
Supplement: Supplementary file 1 [file micromachines-14-00839-s001.zip › micromachines-2295331-supplementary.pdf]

Supporting Information for: "Optical characterization of ALD coated nanoporous alumina structures: Effect of sample geometry or coated layer material"

Ana Laura Cuevas [1], Victor Vega [2], Antonia Dominguez [1], Ana Silvia González [3,*], Víctor M. Prida [3,*] and Juana Benavente [4,*]

[1] Unidad de Nanotecnología, SCBI Centro, Universidad de Málaga, E-29071 Málaga, Spain
[2] Laboratorio de Membranas Nanoporosas, Servicicios Científico-Técnicos, Universidad de Oviedo, E-33006 Oviedo, Spain
[3] Departmento de Física, Facultad de Ciencias, Universidad de Oviedo, E-33007 Oviedo, Spain
[4] Departmento de Física Aplicada I, Facultad de Ciencias, Universidad de Málaga, E-29071 Málaga, Spain
* Correspondence :V.M.P.: vmpp@uniovi.es; J.B.: j_benavente@uma.es

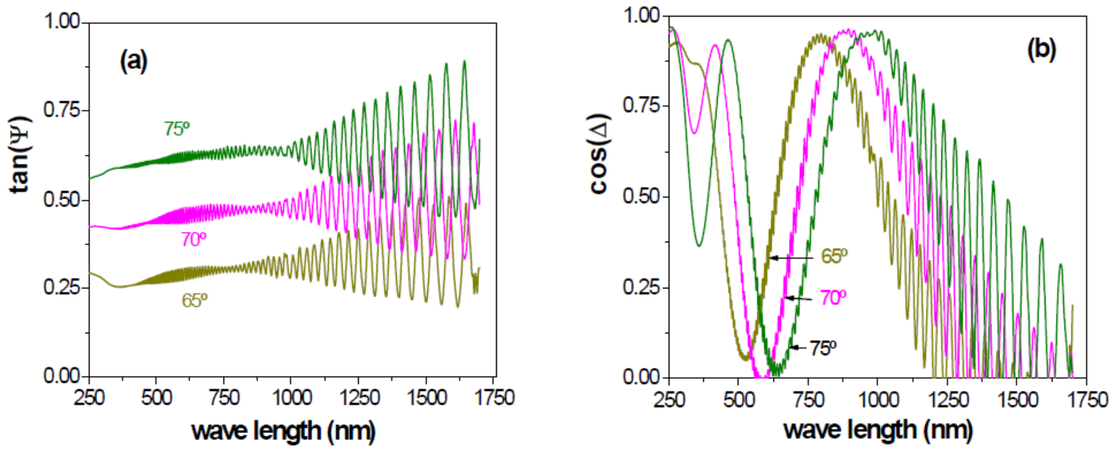

Figure S1: Wavelength dependence for tan(Υ) and cos(Δ) at three light incident angles: 65º (dark yellow lines), 70º (magenta lines) and 75º (green lines) for sample Ox(B)/SiO_2. Without applying data refinement (smoothing)

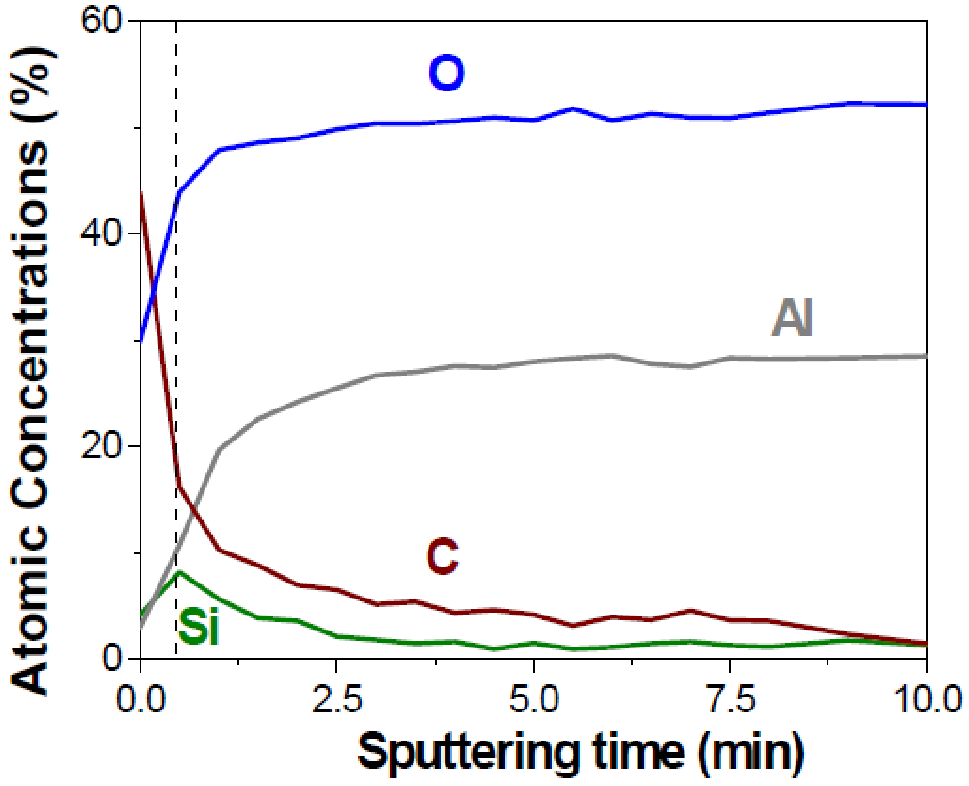

Figure S2: Variation of atomic concentration percentages of the different elements detected on the Ox/SiO_2 sample surface with the XPS sputtering time.

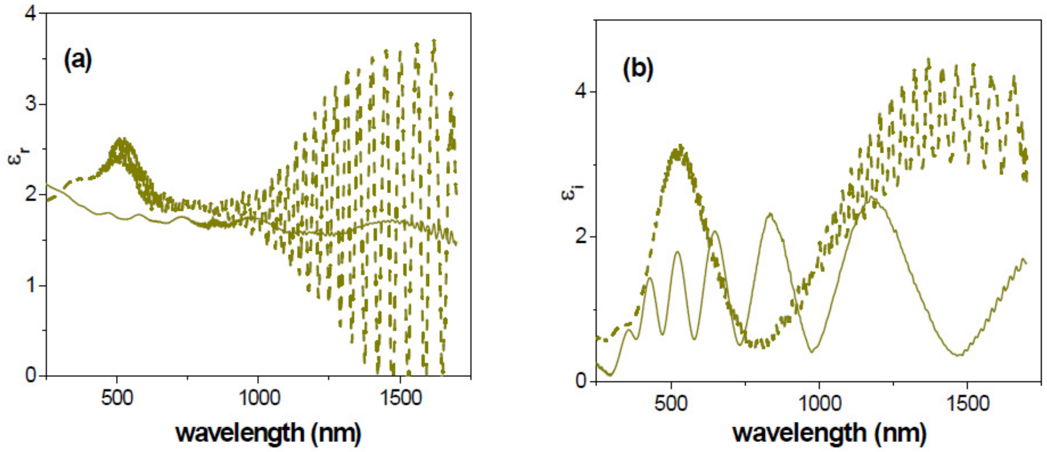

Figure S3: Wavelength dependence of the dielectric constant (real part (a) and imaginary part (b)) for Ox(A)/SiO_2 sample (solid line) and Ox(B)/SiO_2 sample (dashed line) determined for a light incident angle of 65º.

.
